# Evaluation of Point Shear Wave Elastography Using Acoustic Radiation Force Impulse Imaging for Longitudinal Fibrosis Assessment in Patients with HBeAg-Negative HBV Infection

**DOI:** 10.3390/jcm8122101

**Published:** 2019-12-02

**Authors:** Christiana Graf, Antonia Mondorf, Viola Knop, Kai-Henrik Peiffer, Julia Dietz, Julia Friess, Heiner Wedemeyer, Peter Buggisch, Stefan Mauss, Thomas Berg, Michael Rausch, Martin Sprinzl, Hartwig Klinker, Holger Hinrichsen, Jean-Pierre Bronowicki, Sebastian Haag, Dietrich Hüppe, Thomas Lutz, Thierry Poynard, Stefan Zeuzem, Mireen Friedrich-Rust, Christoph Sarrazin, Johannes Vermehren

**Affiliations:** 1 Department of Internal Medicine 1, University Hospital Frankfurt, 60596 Frankfurt, Germany; 2 Department of Gastroenterology, Hepatology and Endocrinology, Hannover Medical School, 30625 Hannover, Germany; 3 Department of Gastroenterology and Hepatology, University Hospital Essen, 45147 Essen, Germany; 4 Institute for Interdisciplinary Medicine IFI, 20099 Hamburg, Germany; 5 Center for HIV and Hepatogastroenterology, 40237 Düsseldorf, Germany; 6 Department of Gastroenterology and Rheumatology, University Hospital Leipzig, 04103 Leipzig, Germany; 7 Practice of Internal Medicine, 10777 Berlin, Germany; 8 Department of Internal Medicine I, University Medical Centre of the Johannes Gutenberg-University, 55131 Mainz, Germany; 9 Department of Medicine II, Division of Hepatology, University Hospital Würzburg, 97080 Würzburg, Germany; 10 Gastroenterology-Hepatology Center, 24105 Kiel, Germany; 11 Hepato-Gastroenterology, Hopital Brabois- CHU Nancy, 54500 Vandoeuvre-les-Nancy, France; 12 Practice of Gastroenterology, 65189 Wiesbaden, Germany; 13 Practice of Hepatology, 44623 Herne, Germany; 14 Infektiologikum, 60596 Frankfurt, Germany; 15 BioPredictive, 75007 Paris, France; 16 Department of Gastroenterology, St. Josefs-Hospital, 65189 Wiesbaden, Germany

**Keywords:** HBV, non-invasive fibrosis assessment, point shear wave elastography, acoustic radiation force impulse imaging, transient elastography, fibrotest, APRI, FIB-4

## Abstract

Background: Accurate assessment of hepatic fibrosis in patients with chronic HBeAg-negative Hepatitis B is of crucial importance not only to predict the long-term clinical course, but also to evaluate antiviral therapy indication. The aim of this study was to prospectively assess the utility of point shear wave elastography (pSWE) for longitudinal non-invasive fibrosis assessment in a large cohort of untreated patients with chronic HBeAg-negative hepatitis B virus (HBV) infection. Methods: 407 consecutive patients with HBeAg-negative HBV infection who underwent pSWE, transient elastography (TE) as well as laboratory fibrosis markers, including fibrosis index based on four factors (FIB-4), aspartate to platelet ratio index (APRI) and FibroTest, on the same day were prospectively followed up for six years. Patients were classified into one of the three groups: inactive carriers (IC; HBV-DNA <2000 IU/mL and ALT <40 U/L); grey zone group 1 (GZ-1; HBV DNA <2000 IU/mL and ALT >40 U/L); grey zone group 2 (GZ-2; HBV-DNA >2000 IU/mL and ALT <40 U/L). Results: pSWE results were significantly correlated with TE (r = 0.29, *p* < 0.001) and APRI (r = 0.17; *p* = 0.005). Median pSWE values did not differ between IC, GZ-1 and GZ-2 patients (*p* = 0.82, *p* = 0.17, *p* = 0.34). During six years of follow-up, median pSWE and TE values did not differ significantly over time (TE: *p* = 0.27; pSWE: *p* = 0.05). Conclusion: Our data indicate that pSWE could be useful for non-invasive fibrosis assessment and follow-up in patients with HBeAg-negative chronic HBV infection.

## 1. Introduction

Chronic hepatitis B virus infection (HBV) is an important global public health issue with considerable morbidity and mortality [1,2].

HBeAg-negative chronic HBV infection, previously termed inactive carrier (IC) state, has become the predominant type of chronic HBV worldwide [3]. It is characterized by persistently low replicative HBV DNA levels, normal ALT and absence of active liver disease. However, some patients with HBeAg-negative chronic hepatitis B (CHB) may present with major fluctuations of viral replication and ALT that may be associated with active necroinflammation or fibrosis with temporary remissions that mimic inactive infection and make it difficult to differentiate from IC [4,5]. These challenging situations have been described as a “Grey Zone” (GZ) [6,7].

Antiviral treatment is generally indicated in patients with persistent or recurrent ALT elevations above the upper limit of normal (ULN, 40 U/L), in association with HBV DNA levels above 2000 IU/mL and/or liver histology showing moderate necroinflammation or fibrosis [8]. For this reason, most clinical practice guidelines still recommend histologic assessment for staging of liver fibrosis and for determining treatment candidacy, in particular when hepatitis B virus (HBV) DNA and/or alanine aminotransferase (ALT) levels are near the threshold for starting therapy [8,9,10]. However, the clinical application of liver biopsy is limited because of its invasive nature associated with potential severe complications. These limitations have stimulated the search for non-invasive approaches [11]. In the past decade, a variety of indirect assessments of liver fibrosis including the measurement of liver stiffness using transient elastography (TE) or point shear wave elastography (pSWE) and biomarkers have been developed. Besides assessment of liver fibrosis before starting antiviral therapy, non-invasive methods may also be used to monitor treatment response and to predict long-term complications associated with CHB infection.

Point shear wave elastography is a relatively new ultrasound based method, which is incorporated within a conventional ultrasound system, thus allowing the visualization of brightness modulation (B-mode) including the ability to select the area to be assessed. In contrast to TE, the accuracy of pSWE is generally not limited by obesity or interfering structures such as vessels or the gall bladder, as the region of interest (ROI) can be positioned manually. Several studies have proven its clinical application through comparisons with other non-invasive fibrosis assessments and the results show that it is a competent diagnostic tool with a higher diagnostic accuracy for advanced fibrosis (F3–F4) than for low-grade fibrosis (F1–F2) [12]. However, its application has mainly been proved by monocentric, retrospective studies and therefore its quality criteria and prognostic values are not well defined. Longitudinal validation in chronic liver diseases, including hepatitis B, is needed in order to develop standardized quality criteria.

The aim of this study was to evaluate pSWE using acoustic radiation force impulse imaging (ARFI) for longitudinal hepatic fibrosis assessment in patients with HBeAg-negative HBV infection who do not require antiviral therapy. pSWE was compared to established non-invasive tests, including TE and biomarkers (aspartate to platelet ratio index, APRI; fibrosis index based on four factors, FIB-4; FibroTest).

## 2. Materials and Methods

### 2.1. Patients

All patients aged 18 and over with chronic HBeAg-negative chronic HBV infection who attended the participating hospitals between June 2009 and December 2018, were considered for enrollment in the study. The diagnosis of chronic HBV infection was proven by the presence of HBsAg and HBV DNA in serum for more than six months. All patients were considered not to be candidates for antiviral treatment at study inclusion based on current guideline recommendations [13,14]. Patients with other viral infections, other causes of liver disease or failure of liver stiffness measurements were excluded from the study.

Patients were classified into three groups based on HBV DNA and ALT levels: (I) inactive carrier (IC) group (patients with HBV-DNA <2000 IU/mL and ALT <40 U/L); (II) grey zone group 1 (GZ-1; patients with HBV-DNA <2000 IU/mL and elevated ALT >40 U/L); (III) grey zone group 2 (GZ-2; patients with HBV-DNA >2000 IU/mL and ALT <40 U/L) [8]. All patients received point shear wave elastography by use of acoustic radiation force impulse (ARFI-) imaging (Acuson S2000; Siemens Healthineers, Mountain View, CA, USA) as well as transient elastography (TE; FibroScan^®^) with the standard probe (M-probe) or the obese probe (XL-probe) (Echosens, Paris, France) at study inclusion and at yearly intervals for 6 years.

Demographic data, including age, gender, ethnicity and body mass index (BMI) were recorded for each individual patient at the time of pSWE and TE measurements. A liver biopsy was performed at the treating physicians’ discretion.

The study and its protocol were prospectively registered with the Clinical Trials register for clinical studies (NCT01090531). The study was performed in accordance with the ethical guidelines of the Declaration of Helsinki and approved by the ethics committee of the Frankfurt University Hospital. All patients gave their written consent before being enrolled in the study.

### 2.2. Biochemical Parameters

The following biological parameters were recorded in each individual patient at the time of pSWE and TE measurements: alanine aminotransferase, aspartate aminotransferase, alpha-2 macroglobulin, apolipoprotein A1, bilirubin, haptoglobin, gamma glutamyl transferase and platelet count.

FibroTest (FT), a commercially available serum fibrosis test (BioPredictive, Paris, France), was performed in each patient and computed on the biopredictive website (www.biopredictive.com) using a patented algorithm that includes alpha-2-macroglobulin, apolipoprotein A1, haptoglobin, gamma glutamyl transferase and total bilirubin, adjusted for age and gender [15].

APRI and FIB-4 scores were calculated for all patients using clinical laboratory data according to the original formula as follows:

APRI = ([AST/upper limit of normal range]/platelet count) × 100,(1)

(2) $$\mathrm{FIB}\text{-}4\;=\;(\mathrm{age}\;\times \;\mathrm{AST})/(\mathrm{platelet}\;\mathrm{count}\;\times \;\sqrt{\mathrm{ALT}}),$$

The cut-offs used for diagnosing significant fibrosis and cirrhosis were those from original publications: FT values were >0.48 and >0.74 respectively [15]; APRI values were <0.5 or ≥1.5 and <1 or ≥2 respectively [16]; FIB-4 values were >1.00 and >3.25 respectively [17].

### 2.3. Liver Stiffness Measurement

pSWE and TE were performed after at least 6 hours of fasting on the same day by the same examiner. The technical details of ARFI imaging have been described previously [18]. In brief, a region of interest (ROI) was placed in the right hepatic lobe on vessel- and tumour-free parenchyma during real-time B-mode imaging. Ten valid acquisitions were obtained in the ROI, with the probe positioned 2.5 cm below the liver capsule. The method uses a transducer that applies short-duration acoustic pulses with a fixed transmitter frequency (2.67 MHz) to generate localized displacements in tissue. These displacements result in shear-wave propagation away from the region of excitation and are tracked by the transducer with ultrasonic correlation-based methods [19]. The results are expressed in meters per second (m/s).

The cut-offs used for diagnosing significant fibrosis and cirrhosis were 1.37 kPa and 1.75 kPa respectively [18].

TE was performed in the right hepatic lobe at the same site as ARFI measurements, as previously described [20]. A total of 10 valid measurements were carried out in each patient. In line with the manufacturer’s recommendations, the examination was considered reliable when at least 60% and an interquartile range of 30% or lower were achieved. The cut-offs used for diagnosing significant fibrosis and cirrhosis were 7.2 kPa and 11.0 kPa [21]. 

Liver fibrosis and necroinflammatory activity were evaluated according to the METAVIR scoring system [22].

### 2.4. Statistical Analysis

Statistical analyses were performed using the R software program (R Foundation for Statistical Computing, Vienna, Austria; version 3.5.1) and IBM SPSS 26.0 statistical software package (SPSS/IBM, Munich, Germany).

For pSWE and TE, the median of the 10 successful measurements obtained in each patient was calculated and used for further analyses. Values of all non-invasive fibrosis assessments were not normally distributed and were therefore expressed as medians. Clinical and laboratory characteristics of patients were expressed as mean ± standard deviation, median and range, as appropriate. Overall, comparisons between pSWE values, TE values, ALT values and biomarkers were performed using the Mann–Whitney *U* test.

Correlations between diagnostic means were assessed using Spearman’s correlation coefficients and their associated probability.

Durbin–Skillings–Mack tests were used to determine overall differences of pSWE, TE, ALT and biomarker values during six years of follow-up.

To identify predictive values of non-invasive fibrosis assessments and patient characteristics at baseline, logistic regression analysis was used. All tests were two-sided and a P value of less than 0.05 was judged to be statistically significant.

## 3. Results

A total of 407 consecutive patients (38% male; mean age 42 ± 12; age range: 19–84) were included in the study. Among these, 28 patients underwent liver biopsy. Based on their METAVIR scores, there were 26 patients with histology showing F0 fibrosis and two patients with F1 fibrosis.

pSWE of the liver failed to achieve 10 valid measurements in 15 patients because of unreliable measurements. In the remaining 392 patients, 10 valid pSWE measurements were successfully obtained from the right lobe of the liver and LS measurements ranged from 0.6 to 1.4 m/s. Median shear wave velocities of 1.0 m/s (0.6–1.3), 1.0 m/s (0.7–1.4), and 1.0 m/s (0.6–1.3) were recorded in IC, GZ-1, and GZ-2 patients, respectively. TE of the liver failed to achieve 10 valid measurements in 14 patients because of unreliable TE-measurements. In the remaining 393 patients, 10 valid TE measurements were obtained. The median liver stiffness was 4.3 kPa (2.6–6.8) in IC, 5.3 kPa (3–8.8) in GZ-1, and 4.6 kPa (2.7–6.6) in GZ-2 patients, respectively. The main baseline patient characteristics of the overall study population, including demographic and biochemical features are listed in Table 1.

**Figure 1:**
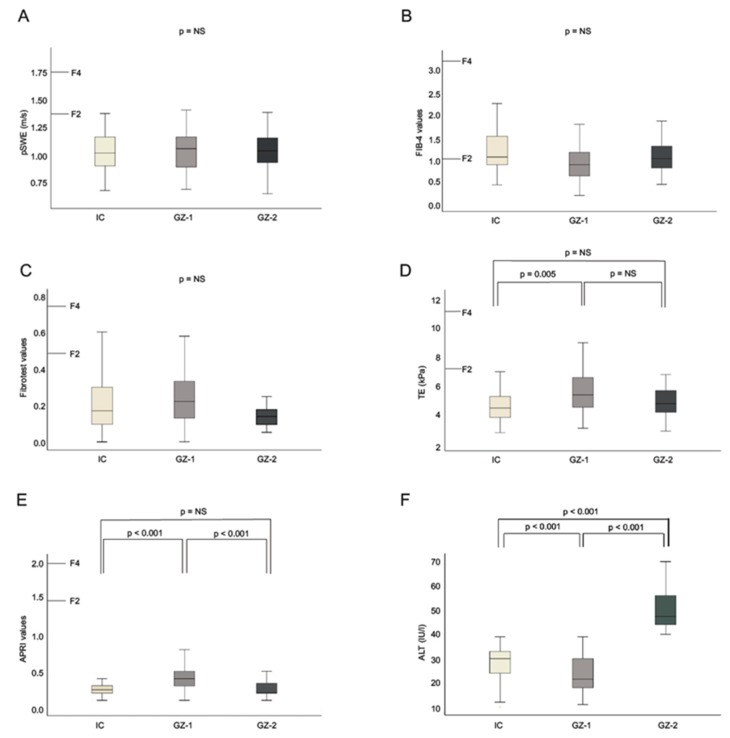
Box plots of point shear wave elastography (pSWE) (**A**), FIB-4 (**B**), FibroTest (**C**), transient elastography (TE) (**D**), APRI (**E**) values and ALT levels (**F**) in inactive carriers (IC), grey zone (GZ)-1 and GZ-2 patients. The top and bottom of the boxes are the first and the third quartiles respectively. The length of the box thus represents the interquartile range (IQR) within which 50% of the values were located. The line through the middle of each box represents the median. The error bars show the minimum and maximum values (range).

For pSWE, FIB-4, and FT, non-invasive liver fibrosis results were not significantly different between the three HBV patient groups (IC vs. GZ-1: pSWE, *p* = 0.82; FIB-4, *p* = 0.81; FT, *p* = 0.37; IC vs. GZ-2: pSWE, *p* = 0.17; FIB-4, *p* = 0.16; FT, *p* = 0.81; GZ-1 vs. GZ-2: pSWE, *p* = 0.34, FIB-4, *p* = 0.42; FT, *p* = 0.51; Figure 1A,B,C).

TE measurements (*p* = 0.005), APRI values (*p* < 0.001) and ALT levels (*p* < 0.001) were significantly higher in GZ-1 patients compared to IC (Figure 1D,E,F).

APRI values were also higher in GZ-1 patients (*p* < 0.001) compared to GZ-2. For the comparison of GZ-2 and IC patients, there was no difference according to TE and APRI (TE, *p* = 0.07; APRI, *p* = 0.32). For all patients, pSWE of the liver was significantly correlated with TE (Spearmans’s rho = 0.29, *p* < 0.001) and APRI (r = 0.17; *p* = 0.005). The overall correlation with aminotransferase levels at baseline and at yearly follow-ups showed statistical significance for TE (AST, *p* < 0.001; ALT, *p* < 0.001) but not for pSWE (AST, *p* = 0.25; ALT, *p* = 0.78).

Of the 407 patients analysed, 255 underwent repeated determinations of pSWE, TE and biomarkers at yearly intervals. Overall, two determinations were performed in *n* = 255 patients, three determinations in *n* = 156, four determinations in *n* = 107, five in *n* = 71, six in *n* = 30 and seven determinations in *n* = 6 patients. This subgroup of patients did not differ from the overall patient cohort for baseline characteristics.

**Figure 2:**
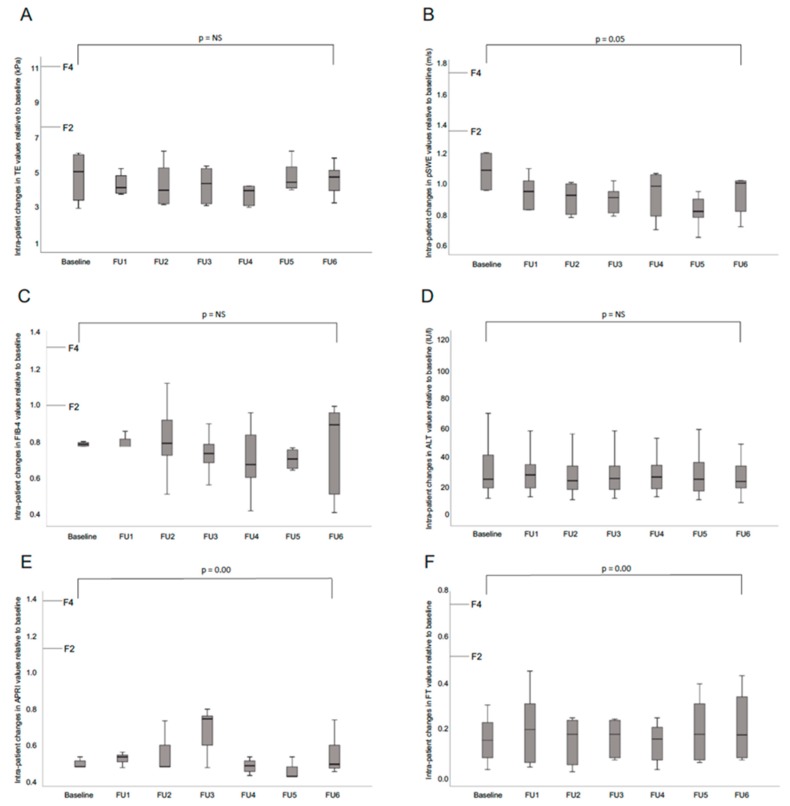
Evolution over time of TE (**A**), pSWE (**B**), FIB-4 (**C**), ALT (**D**), APRI (**E**) and FibroTest (**F**) values of HBeAg-negative chronic hepatitis B (CHB) patients who underwent at least two determinations over time. The top and bottom of the boxes are the first and the third quartiles respectively. The length of the box thus represents the IQR within which 50% of the values were located. The line through the middle of each box represents the median. The error bars show the minimum and maximum values (range).

Intra-patient changes in pSWE and TE, APRI, FIB-4 and FibroTest values at yearly follow up time points relative to baseline are presented in Table S1. As shown in Figure 2A–D, TE, pSWE, FIB-4 and ALT values showed no significant intra-patient changes during up to six years of follow-up. Conversely, the biomarkers APRI and FT showed significant fluctuations over time (Figure 2E,F; Table S1).

There were a total of 16 patients (3.9% of all patients; patient distribution at baseline: IC: 6, GZ-1: 6, GZ-2: 4) who were offered antiviral treatment during follow up because they experienced an increase in HBV DNA levels (*n* = 12) and/or ALT levels (*n* = 9) and qualified for treatment according to the EASL guideline recommendations. These patients had significantly higher TE values (median: 6.0 vs. 4.9 respectively, *p* = 0.03) and HBV DNA levels (median: 710 vs. 198 respectively, *p* = 0.04) but not higher pSWE values at baseline. In a multiple logistic regression analysis, the only independent predictors for therapy initiation during follow-up were higher HBsAg levels and TE values at baseline (Table 2).

13 patients (4%) had elevated pSWE values at first examination suggestive of significant fibrosis (> 1.37 m/s). Liver stiffness values >7.2 kPa could be detected in three of them. 11 patients underwent repeated determinations and pSWE as well as TE values returned below the cut-offs of significant fibrosis during follow-up in all of them.

Beyond that, three patients with normal pSWE and TE values during follow-up had elevated pSWE values in the last examination. ALT levels were normal and HBV DNA levels were low in these patients. Further controls have to follow in order to evaluate these measurements.

## 4. Discussion

In chronic viral hepatitis B, assessment of liver fibrosis is an important parameter to help identifying patients who require appropriate antiviral therapy, not only at first presentation but also during long-term follow-up. In our study, the use of pSWE for follow-up assessment in both IC and GZ patients was compared to TE and biomarkers which had previously been validated for this purpose [13,14,23]. The use of pSWE and similar methods in clinical routine has the advantage of being integrated into a conventional ultrasound system. It can be performed during standard B-mode ultrasound examinations of the liver, which are routinely used for follow-up assessment in patients with chronic liver disease. Besides that, advantages of pSWE include the possibility to choose the positioning of the ROI within the liver, thus avoiding interfering structures such as large blood vessels and bile ducts. Moreover, pSWE is not influenced by obesity or ascites. Therefore, pSWE and other B-mode-based methods are more widely used in non-specialist hospitals and clinical practices.

Our longitudinal study, conducted in a large, well-characterized prospective cohort of consecutive patients with chronic HBeAg-negative HBV infection, assessed the applicability of pSWE for fibrosis assessment in patients who do not require therapy.

Median pSWE values in the entire cohort of patients with HBe-antigen negative CHB were low and comparable to those of healthy individuals as well as to patients with chronic hepatitis C and persistently normal ALT in previously published studies [24,25,26,27].

At baseline, a statistically significant correlation could be detected between pSWE and TE as well as between TE and all biomarker scores, thus demonstrating the comparability of these diagnostic methods.

In the past, TE has been the most widely-studied method for non-invasive fibrosis assessment [28]. However, TE has several limitations, including cost and lack of integration into conventional ultrasound systems, while presence of fibrosis-related factors may lead to an overestimation or underestimation of the extent of fibrosis [29]. Importantly, serum levels of aminotransferases may mimic significantly higher liver stiffness values according to TE, thereby overestimating the extent of liver fibrosis. In our study, TE values were significantly correlated with ALT and AST values at baseline and at yearly follow up, confirming previous observations that TE values may be associated with ALT flares [30].

In contrast, pSWE is a relatively new, cheap and quick imaging tool, and data on its longitudinal use are widely lacking. In a retrospective multicentre study evaluating the influence of aminotransferase levels on liver stiffness assessed by pSWE in European patients with chronic hepatitis B and C it could be demonstrated that moderately elevated ALT values (between 1.1 and 5 x ULN) had no impact on pSWE elastography values [24]. In line with these findings, no significant association between pSWE values and aminotransaminase levels could be found in our prospective study of untreated HBeAg-negative patients (ALT, *p* = 0.55; AST, *p* = 0.81). It is not clearly understood, why TE and not pSWE may be influenced by significant ALT alterations and we do not have a technical explanation for this observation. TE may be prone to more operator-related and patient-related variability [12]. However, the number of patients who received liver stiffness measurements during follow-up may have been too small in our study to draw any definitive conclusions. Indeed, other studies have found the opposite relationship with data suggesting that markedly increased ALT levels may influence pSWE values [31,32].

In our study, mean hepatic shear wave velocities measured with pSWE did not differ significantly between GZ-1 patients, GZ-2 patients and true IC. As pSWE values ranged between 0.7 and 1.4 in our cohort of 54 GZ-1 patients, our data support previous observations which showed that pSWE may be reliable for noninvasive fibrosis assessment in patients with moderately elevated transaminases as compared to other methods [24]. Similarly, FIB-4 and FibroTest scores did not differ significantly between GZ-1 and other CHB patients, suggesting that they are also not influenced by moderately elevated liver enzymes.

In our study, a subgroup of patients had long-term follow-up assessment for up to six years. Interestingly, pSWE, TE and FIB-4 values as well as ALT showed no significant fluctuations over time. In contrast, significant fluctuations could be observed with APRI and FT values. Similar observations have been described in a French study evaluating TE, FT and APRI scores in 201 IC over a median interval of 21.7 months [14]. While serum biomarkers are practical because of their widespread availability and high applicability, interpretation of results may require careful interpretation due to natural fluctuations and the possible impact of comorbid conditions.

In our study, antiviral therapy was started in 16 out of 407 patients during follow-up. Interestingly, these patients showed significantly higher TE values and HBV DNA levels but not higher ALT levels at baseline. Furthermore, besides HBsAg, TE was the only independent method that could predict therapy initiation in a multivariable logistic regression analysis suggesting that it is a highly sensitive non-invasive tool not only to demonstrate the absence of significant fibrosis but also to evaluate and monitor HBe-antigen negative CHB patients longitudinally. While our data indicate that pSWE may be useful to demonstrate the lack of fibrosis progression during long-term follow-up, we failed to show its use for the prediction of therapy initiation, which was only based on HBV DNA and/or ALT flares but not on histology in this study.

## 5. Conclusions

To our knowledge, this is the first study which evaluated the use of pSWE for longitudinal fibrosis assessment in untreated HBeAg-negative patients. Our data demonstrate the clinical applicability of pSWE and its comparability to established non-invasive methods for fibrosis assessment. We propose that pSWE could be useful in addition to HBV DNA and aminotransferase levels for long-term follow-up of patients with HBeAg-negative HBV infection.

Our study is limited by the small number of subjects tested, who developed significant fibrosis as well as the small number of patients who received liver biopsies. Therefore, validation of the accuracy of pSWE for diagnosing significant fibrosis and cirrhosis according to histological results was not possible. Yet, we could demonstrate the comparability of pSWE to other already established methods for longitudinal liver stiffness measurement in IC patients who do not require antiviral therapy.

Future studies are needed to assess the use of combining pSWE with other fibrosis markers to better predict clinical endpoints including the need for antiviral therapy during follow-up.

## Figures and Tables

**Table 1:** Baseline patient characteristics according to their status.

Characteristics	HBeAg-Negative Total (*n* = 407)	IC (*n* = 308)	GZ-1 (*n* = 54)	GZ-2 (*n* = 45)
**Patient age (years) Mean** **±** **SD**	42 ± 12	42 ± 12	42 ± 14	38 ± 9
**Male gender, *n* (%)**	157 (38%)	104 (32.5%)	29 (48%)	19 (41.3%)
**Caucasian Ethnicity, *n* (%)**	212 (52.1%)	164 (53.2%)	25 (46%)	24 (52.2%)
**BMI (kg/m^2^), mean** **±** **SD**	25.9 ± 5.0	25.8 ± 4.9	27.5 ± 5.1	25 ± 5.1
**AST (IU/L) mean** **±** **SD**	24.7 ± 8.0	23.2 ± 5.9	35 ± 12	22.9 ± 5.8
**ALT (IU/L) mean** **±** **SD**	27.4 ± 13.4	23.4 ± 7.4	54 ± 13	23.7 ± 7.4
**Median HBsAg (IU/mL)**	1261	1125	940	2090
**Median HBV DNA (IU/mL)**	210	150	157	6750
**Median TE**	4.8	4.7	5.3	4.9
**Median ARFI**	1.1	1.1	1.1	1.1
**Median APRI score**	0.3	0.3	0.4	0.3
**Median FIB-4 score**	0.9	0.9	0.9	0.8
**Median FibroTest score**	0.2	0.2	0.2	0.2
**Fibrosis stage (METAVIR), *n* (%)**	28 (6.8%)	15 (4.9%)	5 (9.3%)	8 (17.8%)
**F0**	26	14	4	8
**F1**	2	1	1	0
**F3**	0	0	0	0
**F4**	0	0	0	0

ALT, alanine aminotransaminase; APRI, aspartate to platelet ratio index; ARFI, acoustic radiation force impulse imaging; AST, aspartate aminotransaminase; BMI, body mass index; FIB-4, fibrosis index based on four factors; GGT, gamma-glutamyl transpeptidase; HBV, hepatitis B virus; IC, inactive carrier; SD, standard deviation; TE, transient elastography.

**Table 2:** Assessment of the logistic regression analysis of independent factors associated with therapy initiation in chronic HBV patients.

Variable	B	SE	Wald	df	95% CI of Exp (b)		*p*
					Upper	Lower	
**Age**	0.05	0.04	2.09	1	0.98	1.13	0.15
**Gender**	−0.52	0.82	0.39	1	0.12	2.99	0.53
**Ethnicity**	0.63	0.34	3.50	1	0.97	3.67	0.06
**HBsAg**	0.00	0.00	6.41	1	1.00	1.00	0.01
**HBV DNA**	0.00	0.00	0.43	1	1.00	1.00	0.51
**ALT**	0.01	0.03	0.24	1	0.96	1.07	0.62
**TE**	0.71	0.30	5.45	1	1.12	3.69	0.02
**pSWE**	1.41	2.04	0.49	1	0.08	230.37	0.48
**APRI**	1.77	2.77	0.41	1	0.03	1326.77	0.52
**FIB-4**	0.00	0.07	0.00	1	0.87	1.15	0.98
**Fibrotest**	−6.85	4.10	2.78	1	0.00	3.31	0.10
**Constant**	−12.35	3.58	11.94	1			0.00

ALT, alanine aminotransaminase; APRI, aspartate to platelet ratio index; FIB-4, fibrosis index based on four factors; HBsAg, hepatitis B antigen; HBV DNA, hepatitis B virus deoxyribonucleic acid; pSWE, point shear wave elastography; TE, transient elastography.

## Supplementary Materials

The following are available online at https://www.mdpi.com/2077-0383/8/12/2101/s1, Table S1: Median (IQR) intra-patient TE, pSWE, APRI, FIB-4 and Fibrotest values changes at different time points relative in chronic HBV patients with at least two determinations of non-invasive methods over time.
