# Supplementary material for: Evaluation of Point Shear Wave Elastography Using Acoustic Radiation Force Impulse Imaging for Longitudinal Fibrosis Assessment in Patients with HBeAg-Negative HBV Infection
Source: J Clin Med. 2019 Dec 2;8(12):2101. doi: 10.3390/jcm8122101 (PMC6947378; doi:10.3390/jcm8122101)
Supplement: Supplementary file 1 [file jcm-08-02101-s001.pdf]

Table S1. Median (IQR) intra-patient TE, pSWE, APRI, FIB-4 and Fibrotest values changes at different time points relative in chronic HBV patients with at least two determinations of non-invasive methods over time.

Time Point	Patients	TE (kPa)		pSWE (m/s)		APRI		FIB-4		Fibrotest	
**FU1**	$n = 255$	−0.10	$p = 0.82$	0.00	$p = 0.65$	+0.01	$p = 0.67$	+0.05	$p = 0.00$	+0.01	$p = 0.16$
**FU2**	$n = 156$	−0.01	$p = 0.88$	−0.02	$p = 0.62$	−0.01	$p = 0.82$	−0.01	$p = 0.01$	−0.03	$p = 0.26$
**FU3**	$n = 107$	−0.06	$p = 0.91$	−0.01	$p = 0.66$	−0.01	$p = 0.73$	+0.05	$p = 0.05$	−0.02	$p = 0.17$
**FU4**	$n = 71$	−0.20	$p = 0.72$	0.00	$p = 0.85$	−0.01	$p = 1.0$	+0.02	$p = 0.12$	−0.03	$p = 0.16$
**FU5**	$n = 30$	−0.09	$p = 0.77$	−0.07	$p = 0.09$	−0.03	$p = 0.03$	+0.02	$p = 0.22$	+0.03	$p = 0.00$
**FU6**	$n = 6$	−0.51	$p = 0.27$	−0.04	$p = 0.05$	−0.05	$p = 0.00$	−0.04	$p = 0.30$	+0.01	$p = 0.00$

APRI, aspartate to platelet ratio index; FIB-4, fibrosis index based on four factors; FU, Follow up; pSWE, point shearwave elastography; TE, transient elastography.
